# Micro‐CT Evaluation of Dentin Preservation by ProTaper Gold and VDW.Rotate in Oval Mandibular Incisors

**DOI:** 10.1155/tswj/8289243

**Published:** 2026-01-22

**Authors:** Wesley Viana de Sousa, Marina da Cunha Isaltino, Christianne Velozo, Silmara de Andrade Silva, Luiza de Almeida Souto Montenegro, Hugo Victor Dantas, Frederico Barbosa de Sousa, Diana Albuquerque

**Affiliations:** ^1^ Dental College of Pernambuco, Department of Restorative Dentistry and Endodontics, University of Pernambuco, Recife, Brazil, ufpe.br; ^2^ Health Sciences Center, Department of Morphology, Federal University of Paraíba, João Pessoa, Paraíba, Brazil, ufpb.br

**Keywords:** mandibular incisors, NiTi endodontics instruments, ProTaper Gold, root canal preparation, VDW.Rotate

## Abstract

**Introduction:**

The success of endodontic treatment depends on the effective chemomechanical preparation of the root canal system, ensuring proper shaping, cleaning, and disinfection. Additionally, preserving pericervical dentin may improve the long‐term prognosis of the tooth. The aim of this study was to compare the performance of two nickel‐titanium rotary systems, ProTaper Gold (PTG) (Dentsply Sirona, Ballaigues, Switzerland) and VDW.Rotate (VDWR) (VDW GmbH, Munich, Germany), in preparing mandibular incisors, focusing on pericervical dentin preservation.

**Methods and Materials:**

A total of 20 mandibular incisors with oval‐shaped root canals (Vertucci Type I) were selected, disinfected, and scanned by micro‐CT. After randomization, the PTG and VDWR systems were used for canal preparation (*n* = 10). Preoperative and postoperative images were processed to measure volume, surface area, structure model index, and untouched walls. Irrigation with 2.5% NaOCl and 17% EDTA was standardized. Data were analyzed using the Student *t*‐test, Welch *t*‐test, or Mann–Whitney *U* test, with *α* = 5*%*.

**Results:**

Both systems exerted similar effects on proximal wall diameter reduction. The PTG system promoted significantly greater increases in volume (PTG: 5.50%; VDWR: 3.57%) and surface area (PTG: 34.79%; VDWR: 26.93%) compared with that of VDWR (*p* < 0.05). The percentage of unprepared areas was (PTG: 1.57%; VDWR: 2.33%).

**Conclusion:**

VDWR was associated with reduced dentin removal versus PTG in vitro, but clinical superiority still needs to be proven.

## 1. Introduction

The success of endodontic treatment depends on the preservation of the tooth, the maintenance of its functionality, the absence of symptoms, and the integrity of periradicular tissues [1–3]. Chemomechanical preparation plays a key role in achieving these goals by reducing the bacterial load within the root canal system to levels insufficient to sustain or induce apical periodontitis [4–6]. Despite technological advances, achieving optimal cleaning and shaping remains a challenge. Micro‐computed tomography (micro‐CT) studies have shown that a relevant percentage of canal walls can remain untouched, potentially harboring tissue or biofilms that compromise disinfection [6].

Both ProTaper Gold (PTG) and VDW.ROTATE (VDWR) are heat‐treated nickel–titanium (NiTi) systems that use continuous rotary motion [7–10]. PTG, extensively evaluated in the literature for its shaping ability [10–14], has a convex triangular cross section designed for efficient dentin removal, whereas VDWR features an S‐shaped cross section with manufacturer claims of “anatomy‐preserving” shaping [12, 15]. These distinct geometries may influence cutting efficiency, canal catering, and dentin preservation, especially in delicate regions such as the pericervical dentin [15, 16].

Although PTG has been widely studied in diverse anatomies [17–20], evidence regarding VDWR—particularly in mandibular incisors—is scarce. Its inclusion in the present comparison is justified by its conservative design philosophy and claims of maintaining original canal geometry, which, if proven effective in narrow and long‐oval anterior canals, could support more dentin‐preserving strategies. Mandibular incisors present a relevant clinical model due to their narrow cross section, thin dentin walls, and high susceptibility to structural weakening after preparation. Preservation of pericervical dentin in these teeth directly impacts postendodontic restoration prognosis and fracture resistance, influencing long‐term treatment outcomes [15, 16].

Thus, this in vitro study aimed to compare, by micro‐CT, the shaping ability of the PTG and VDWR systems in mandibular incisors. The null hypothesis tested was that there would be no significant difference in the mechanical preparation of the root canal between these two NiTi rotary file systems.

## 2. Material and Methods

### 2.1. Sample Size Calculation

The Ethics Committee on Research Involving Humans of the University of Pernambuco (UPE) approved the study (Protocol 076805/23). The sample size was calculated using the G∗Power 3.1 software (Heinrich Heine Universität, Düsseldorf, Germany) and was based on a previous study [21]. Sample size calculation assuming an alpha error of 0.05 and power of 80% resulted in eight specimens per group. To account for possible sample losses during the study, 10 samples were included in each group.

### 2.2. Sample Selection and Characterization

Tooth specimens were selected from a collection of 150 extracted human mandibular incisors, Vertucci Type I [22], extracted for reasons not related to this study. The teeth were disinfected in a 0.1% thymol solution for 24 h and stored in purified filtered water for no longer than 30 days to avoid structural alterations in dentin. For selection, each tooth was first examined under a dental operating microscope at 10× magnification (Alliance, São Paulo, SP, Brazil) and radiographed in the mesiodistal and buccolingual directions. Teeth with endodontic treatment and roots with caries, vertical or horizontal fractures, internal resorption defects, immature apices, moderate to severe apical curvature, or two canals were excluded. Unrestored or moderately restored straight mandibular incisors with a single root and a single canal were included. Teeth with moderate to severe apical curvature, defined as Schneider′s angle ≥ 10°, were also excluded.

After radiographic evaluation and application of the predefined inclusion and exclusion criteria, the samples were scanned with the Bruker SkyScan 1172 high‐resolution micro‐CT system (Bruker SkyScan, Kontich, Belgium) to generate three‐dimensional (3D) models of the root canals for qualitative assessment of their anatomy configuration [21]. The average preoperative canal volume and dimensions were calculated from baseline micro‐CT scans and are presented in Table 1. The allocation sequence was generated by computer software (http://random.org/), and group assignment was concealed in sealed opaque envelopes. All preoperative and postoperative micro‐CT analyses were performed by a single calibrated operator blinded to the group allocation.

**Table 1:** Pre‐instrumentation canal volume and mesiodistal diameter in mandibular incisors.

	**ProTaper Gold mean ± SD**	**VDW.Rotate mean ± SD**	**p**
Canal volume (mm^3^)	2.59 ± 0.85	3.43 ± 1.17	0.083^a^
Diameter–mesial (mm)	2.27 ± 0.22	2.30 ± 0.31	0.896^a^
Mesiodistal diameter–distal (mm)	2.25 ± 0.39	2.27 ± 0.19	0.893^a^

*Note:* Statistical significance at the 5.0% level.

^a^ Mann–Whitney *U* test.

### 2.3. Root Canal Preparation

A total of 20 teeth were selected and randomized to the two experimental groups (*n* = 10): PTG and VDWR. One experienced endodontist previously trained in the test systems performed all preparations. Each set of instruments was used to prepare two root canals according to the manufacturers′ recommendations.

The teeth were mounted on a dental mannequin in a mandibular jaw under rubber dam isolation to reproduce the clinical conditions. All procedures were performed under an operating microscope. Conventional coronal access was performed, and the same irrigation protocol was used in the two groups during canal preparation. The irrigant solution (2.5% NaOCl) was administered at room temperature (≈22°C–25°C) using a NaviTip 30‐gauge needle tip (Ultradent, Joinville, SC, Brazil) at each instrument change (2 mL), up to 2 mm short of the working length (WL), and the total volume was standardized at 10 mL per canal. The smear layer was removed by irrigation with 5 mL of 17% ethylenediaminetetraacetic acid (EDTA) (Maquira, São Paulo, Brazil) (2 min), followed by final irrigation with 5 mL of 2.5% NaOCl (2 min). During canal preparation, ultrasonic, sonic, and mechanical activation methods were not used to avoid introducing confounding variables and to ensure that the observed differences reflected only the mechanical effects of the rotary systems on dentin removal. However, the final irrigation protocol followed a standardized passive ultrasonic irrigation (PUI). After shaping, each canal received 5 mL of 17% EDTA, 5 mL of 2.5% NaOCl, and a final rinse with distilled water. Both NaOCl and EDTA were activated using an Irrisonic E1 tip, with no cutting power (Helse, São Paulo, Brazil) from 2 mm short of the WL, activated by the Advance View ultrasonic unit (Microdont, São Paulo, Brasil) at 20% power, avoiding contact with the root canal walls.

Patency of the apical foramen was maintained throughout the procedures with a #10 K‐file (Dentsply Sirona, Ballaigues, Switzerland). When the tip of the file became visible at the main foramen, the WL was established 1.0 mm short of this point. No coronal flaring was performed. A glide path to the WL was created using a #15 K‐file, which was selected instead of ProGlider to standardize procedures across both groups, given that all canals were straight and narrow. Before the shaping procedures, each root was sealed apically with a thin layer of light‐cured resin (Whitegold Protector Blue, Dentsply Sirona) to simulate a closed system. After instrumentation, the canals were dried with sterile absorbent paper points (Cell Pack, Tanari, Manaus, Brazil) corresponding to the final instrument size. The shaping procedures for each instrumentation system were then performed according to their respective clinical protocols, as detailed in the following sections.

#### 2.3.1. PTG

The teeth were instrumented in short‐amplitude movements until the WL was reached. The S1 (18/0.02), S2 (20/0.04), F1 (20/0.07), F2 (25/0.08), and F3 (30/0.09) instruments were used sequentially, powered by an X‐Smart Plus motor (Dentsply), in continuous rotation at 300 rpm. Torque was set at 5.2 N·cm (S1), 1.5 N·cm (S2), and 3.1 N·cm (F1, F2, and F3), according to the manufacturer′s instructions. The sequence was performed up to F3 (30/0.09) following the standard clinical protocol recommended by the manufacturer, even though the taper differs from the last instrument in the VDWR sequence. This choice was made to reproduce real clinical use, acknowledging that differences in taper may result in variations in dentin removal.

#### 2.3.2. VDWR

In this group, the rotary system was also operated in continuous rotation at 2.0 N·cm torque and 350 rpm, as recommended by the manufacturer. The (15/0.04), (20/0.05), (25/0.06), and (30/0.04) instruments were used sequentially. The instrument was advanced and retracted with slight apical pressure until the WL was reached.

After three in–out movements, the instrument was removed from the canal and cleaned, and apical patency was confirmed with a hand #15 K‐file. The canal was irrigated with 2.5% NaOCl and the instrument reutilized until it reached the WL. The sequence was completed with the 30/0.04 instrument, which represents the standard final size recommended by the manufacturer. Although its taper differs from the PTG F3 instrument (30/0.09), this choice was maintained to reproduce clinical practice, acknowledging that taper variability may influence dentin removal outcomes.

### 2.4. Micro‐CT and 3D Model Analysis

The specimens were scanned before and after instrumentation using the SkyScan 1172 micro‐CT system (Bruker micro‐CT, Kontich, Belgium) at the Laboratory of Microscopy and Biological Imaging (LAMIB), Department of Morphology, Federal University of Paraíba (UFPB). The preoperative and postoperative scans were performed under identical conditions, with the acquisition parameters set at 100 kV, 100 *μ*A, 0.6° rotation step, 360° rotation, isotropic resolution of 26.80 *μ*m, and an aluminum/copper filter. Each scan lasted approximately 15 min at room temperature, ensuring reproducibility. All raw data were reconstructed using the NRecon software (Bruker microCT) at an exposure time of 432 ms, with rotation of 0.04, framing average of 4, ring artifact correction of 8, beam hardening correction of 6%, smoothing of 0, and CS‐to‐image conversion values ranging from 0.0 to 0.04. These parameters were uniformly applied in both scanning sessions to ensure methodological consistency. A final reconstruction was conducted in NRecon (Version 1.5.23), with smoothing set at 5, ring artifact correction at 6, and beam hardening correction at 25% to refine and standardize the final images. The same thresholding principles were used in all reconstruction steps to prevent variability arising from software adjustments rather than experimental factors.

To assess volume changes and to calculate the structure model index (SMI), which quantifies alterations in canal shape, a region of interest extending from the cementoenamel junction to the apex was then defined in CTAnalyser (Version 1.17.7.2, Bruker) following the manufacturer′s guidelines (MN110). Identical binarization thresholds and smoothing parameters were consistently applied across all samples to ensure comparability. Although the SMI is a global descriptor and does not identify dentin removal by thirds, it was included as a complementary measure since overall canal geometry changes may impact the mechanical behavior of the filled canal and the long‐term resistance of the root to fracture. To confirm measurement reliability, each volume, surface area, and SMI measurement was performed twice at a 1‐week interval by the same operator. All postoperative analyses were performed by a calibrated examiner blinded to the group allocation, and the intraclass correlation coefficient exceeded 0.9, indicating excellent consistency [23]. Postoperative 3D models were also generated using the same reconstruction protocol. Based on these standardized procedures, any differences in canal morphology observed are attributable to the experimental variables rather than variations in scanning or reconstruction methodology.

### 2.5. Statistical Analysis

Data normality and homoscedasticity were assessed using the Shapiro–Wilk and Levene tests, respectively. The significance level was set at *p* < 0.05 for all statistical analyses. Permutation‐based analysis of variance was used to confirm the baseline homogeneity of the groups regarding volume and surface area. Depending on data distribution and variance characteristics, different statistical tests were applied for comparison of the groups: Student′s *t*‐test for normally distributed data with equal variances, Welch′s *t*‐test for normally distributed data with unequal variances, and Mann–Whitney *U* test for non‐normally distributed data. For paired assessments, the paired Student *t*‐test was used for normally distributed data and the paired Wilcoxon test for non‐normally distributed data. The margin of error adopted in the statistical tests was 5%. Statistical calculations were performed using IBM SPSS (Version 23) (IBM SPSS, Inc., Chicago, Illinois, United States).

## 3. Results

**Figure 1:**
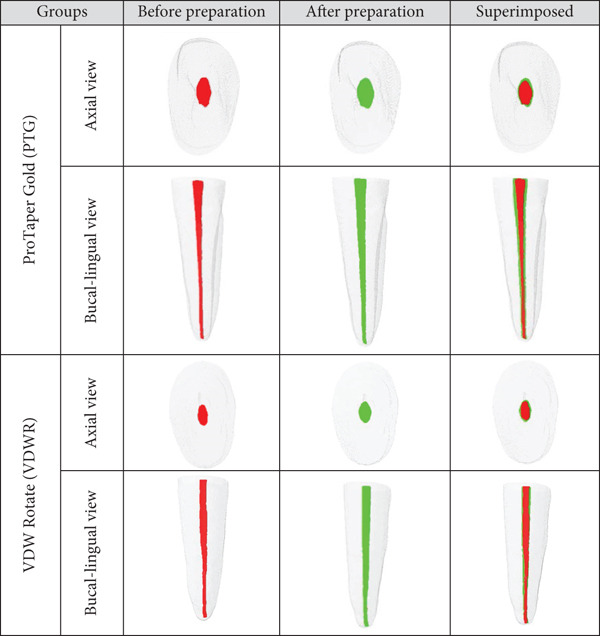
Buccal‐lingual and axial view of three‐dimensional (3D) reconstruction before (in red) and after (in green) preparation with the ProTaper Gold and VDW.Rotate system of mandibular incisors.

All findings are numerically presented in Tables 2 and 3, with 3D visualizations shown in Figure 1. A total of 20 teeth (10 per group) were evaluated, and baseline homogeneity was confirmed for canal morphology before instrumentation (*p* > 0.05) (Table 2).

**Table 2:** Statistical comparison of volume, surface area, SMI, and percentage of unprepared areas before (A) and after (B) preparation between the two groups.

		**ProTaper Gold (PTG) mean ± DP median (P25; P75)**	**VDW.Rotate (VDWR) mean ± DP median (P25; P75)**	**p**
Volume	A	2.59 ± 0.85 2.51 (1.81; 3.41)	3.43 ± 1.17 3.72 (2.73; 4.01)	*p* = 0.083^a^
	B	5.50 ± 0.88 5.33 (4.73; 6.39)	5.20 ± 1.13 5.12 (4.41; 6.29)	*p* = 0.519^a^
	% Increase	127.60 ± 66.26 99.05 (80.83; 166.62)	67.89 ± 68.59 46.08 (25.45; 82.07)	*p* = 0.007^∗b^
	*p* value	*p* = 0.002^∗c^	*p* < 0.001^∗d^	

Surface area	A	27.96 ± 6.02 28.05 (23.26; 30.46)	31.85 ± 6.55 32.22 (25.20; 37.16)	*p* = 0.184^a^
	B	34.79 ± 4.94 34.42 (30.64; 37.03)	35.60 ± 5.76 37.59 (30.74; 40.91)	*p* = 0.739^a^
	% Increase	26.45 ± 13.25 23.39 (16.65; 35.02)	13.04 ± 10.36 8.52 (5.59; 20.22)	*p* = 0.021^∗a^
	*p* value	*p* < 0.001^∗d^	*p* = 0.001^∗d^	

SMI	A	1.76 ± 0.51 1.55 (1.33; 2.30)	1.72 ± 0.57 1.54 (1.38; 2.16)	*p* = 0.876^a^
	**B**	2.36 ± 0.36 2.29 (2.06; 2.69)	2.11 ± 0.41 2.07 (1.78; 2.40)	*p* = 0.169^a^
	% Increase	38.27 ± 18.14 42.39 (17.14; 53.70)	30.69 ± 41.62 17.11 (13.04; 29.55)	*p* = 0.128^b^
	*p* value	*p* < 0.001^∗d^	*p* = 0.002^∗c^	

% Unprepared areas (after**)**		1.57 ± 0.78 1.65 (1.15; 2.32)	2.33 ± 0.50 2.31 (2.11; 2.61)	*p* = 0.019^∗a^

∗Significant difference at the 5.0% level.

^a^Student′s *t*‐test with equal variances.

^b^Mann–Whitney test.

^c^Paired Wilcoxon test.

^d^Paired Student′s *t*‐test.

**Table 3:** Statistical dentin thickness analysis of the mesial and distal, before (A) and after (B) preparation and the reduction (%) between the two groups.

		**ProTaper Gold mean ± SD median (P25; P75)**	**VDW.Rotate mean ± SD median (P25; P75)**	**p**
Mesial Diameter	A	2.27 ± 0.22 2.35 (2.03; 2.49)	2.30 ± 0.31 2.29 (2.09; 2.59)	*p* = 0.896^a^
	B	2.19 ± 0.19 2.22 (1.99; 2.33)	2.16 ± 0.24 2.14 (2.02; 2.34)	*p* = 0.793^b^
	% Reduction	3.85 ± 5.15 0.83 (0.00; 7.65)	5.91 ± 4.98 3.86 (2.60; 12.48)	*p* = 0.246^a^
	*p* value	*p* = 0.063^d^	*p* = 0.004^∗d^	

Distal Diameter	A	2.25 ± 0.39 2.24 (1.98; 2.66)	2.27 ± 0.19 2.31 (2.08; 2.44)	*p* = 0.893^c^
	B	2.19 ± 0.33 2.22 (1.98; 2.45)	2.21 ± 0.22 2.22 (2.00; 2.40)	*p* = 0.851^b^
	% Reduction	2.58 ± 5.07 1.09 (0.00; 2.37)	2.76 ± 3.84 2.78 (0.00; 5.69)	*p* = 0.622^a^
	*p* value	*p* = 0.031^∗d^	*p* = 0.054^e^	

∗ Statistical significance at the 5.0% level.

^a^ Mann–Whitney *U* test.

^b^ Student′s *t*‐test with equal variances.

^c^ Student′s *t*‐test with unequal variances.

^d^ Wilcoxon signed‐rank test.

^e^ Paired Student′s *t*‐test.

After instrumentation, significant differences between PTG and VDWR were observed for canal volume (mean increase: 5.50% [95% CI, 4.83–6.16] vs. 3.57% [95% CI, 2.92–4.23]; Cohen′s *d* = 0.82, large effect size) and surface area (34.79% [95% CI, 29.44–40.14] vs. 26.93% [95% CI, 22.08–31.78]; Cohen′s *d* = 0.75). In contrast, there were no statistically significant differences in SMI (38.27 ± 18.14 for PTG vs. 30.69 ± 41.62 for VDWR; *p* = 0.128; Cohen′s *d* = 0.22) or in the percentage of unprepared areas (PTG: 9.43% vs. VDWR: 12.22% and *p* > 0.05). Although these values are lower than typically reported in the literature (6%–14%), this discrepancy may be explained by methodological factors, including the selection of narrow and straight canals, continuous NaOCl irrigation during shaping, and the potential masking of untouched walls by residual debris.

Regarding cervical diameter changes (Table 3), distal reduction reached statistical significance for PTG (mean change: 0.28 mm; *p* = 0.031), whereas mesial reduction did not. This asymmetry likely reflects the anatomical differences of oval canals, in which the distal wall is more prone to dentin removal due to instrument trajectory. All shaping procedures were performed by a single experienced operator (> 10 years of clinical practice). Although no interoperator variability was assessed, reproducibility was supported by prior operator training with both systems. Importantly, postoperative micro‐CT analyses were blinded to the experimental groups, ensuring unbiased measurement.

## 4. Discussion

A total of 20 extracted mandibular incisors, selected for their oval and long‐oval canal anatomy [21, 24], were used to analyze by micro‐CT the shaping ability of two NiTi systems (PTG and VDWR). The anatomical morphology of these teeth not only facilitates laboratory analysis and ensures consistent outcomes but also makes them a suitable model for studying root canal preparation, reinforcing the importance of this in vitro study [25]. Micro‐CT is the gold standard for analyzing internal morphology and is widely used to assess endodontic instrument performance [26], which supports its use in this study.

Comparing well‐established systems with newly introduced instruments can reveal potential advantages for critical parameters of treatment, while providing relevant information on their expected clinical performance. Studies such as those conducted by Santa‐Rosa et al. [27], Zinge and Patil [28] and Silva et al. [14] demonstrated the varying impacts of different rotary and reciprocating systems on root canal preparation, emphasizing the importance of selecting the appropriate system based on clinical requirements. Although previous studies have compared systems with different kinematics, this study focused on two rotary systems to control motion parameters, considering the following criteria: increase in volume and surface area, SMI, percentage of unprepared areas, and percentage of removed dentin from the mesial and distal surfaces.

Buchanan [29] introduced the concept of variable taper endodontic instruments, noting that large taper instruments cause greater dentin removal, particularly in the cervical region. This fact may explain the greater dentin removal observed in this study for PTG compared to VDWR. The PTG system uses a sequential approach consisting of multiple instruments of varying dimensions and geometries to shape the root canal [16, 19]. In the present study, preparation was limited to F3 (30/0.09), an instrument with a convex triangular cross section, whereas the SX instrument was deliberately excluded to minimize pericervical dentin removal [30].

The VDWR system, which is characterized by its S‐shaped cross section, also operates in continuous clockwise rotation and has been specifically designed to preserve the natural canal anatomy, as demonstrated in recent studies [12, 15]. These attributes, in conjunction with minimal canal transportation [31] align with the goal of preserving pericervical dentin, which is critical for maintaining the structural integrity and long‐term prognosis of the tooth [15]. In the VDWR group, preparation was performed up to the 30/0.04 instrument to standardize the tip instrumentation characteristics. It is important to highlight that, although both systems share the same apical size (#30), their final taper profiles differ considerably (PTG F3 = 30/0.09; VDWR 30/0.04). This taper discrepancy represents a significant methodological variable that makes direct comparison challenging, as it is not possible to determine whether the observed differences in dentin removal are attributable to taper or to instrument design and cutting efficiency. Thus, these findings should be interpreted considering this methodological constraint.

Excessive removal of dentin, particularly in the coronal and apical regions, can compromise tooth integrity and increase the risk of periodontal complications. Thus, techniques that minimize excessive dentin removal are essential [16, 30]. Consistent with this principle, the present study demonstrated significant differences in volume and surface area, with PTG removing more dentin than VDWR (*p* < 0.05), reinforcing the superior ability of the latter in preserving dentin integrity. Although statistically significant, the difference in volume increase between PTG (5.50%) and VDWR (3.57%) represents a small numerical variation that is unlikely to have clinical relevance. Similarly, the difference in unprepared areas between groups (1.57% vs. 2.33%) should also be interpreted as clinically negligible despite reaching statistical significance. Therefore, the null hypothesis was partially rejected since differences were found in volume and surface area, whereas SMI or unprepared areas did not differ significantly.

Another methodological aspect that must be considered is that all shaping procedures were performed by a single experienced operator. Although this approach improves standardization and minimizes procedural variability, it also introduces operator‐dependent influence as a potential source of bias. The absence of interoperator assessment limits the generalizability of the findings, as different clinicians may apply different pressure, amplitude, or tactile control during instrumentation. Consequently, these findings should be interpreted with measured consideration in terms of their reproducibility across different operators.

In the present study, the mean percentage of unprepared areas after canal preparation with the PTG system ranged from 1.15% to 2.32%. Previous studies investigating mesial and distal roots of molars reported a percentage of unprepared areas ranging from 6% to 14% when PTG was used [10, 14], demonstrating its efficient canal shaping ability in more complex anatomies. The lower percentages in our sample are probably related to the less complex and straighter anatomy of Vertucci Type I mandibular incisors, which facilitates more complete contact between instruments and dentin walls. Although mandibular incisors provide a suitable and reproducible model for micro‐CT analysis due to their narrow and long‐oval morphology, the anatomical homogeneity of the present sample restricts the generalizability of the findings. In clinical practice, mandibular incisors often exhibit bifurcations, secondary canals, or subtle curvatures that may influence instrument behavior and dentin‐removal patterns. Consequently, the performance of PTG and VDWR in more anatomically complex scenarios may differ from the trends observed in this controlled in vitro model.

Conversely, the percentage of unprepared areas obtained for the VDWR system ranged from 2.11% to 2.61%. This finding is consistent with its conservative taper, although it should be interpreted with caution since no direct measurement of pericervical dentin thickness was performed in this study. Therefore, preservation of this region cannot be confirmed based solely on volumetric analysis.

Barbosa et al. [32] explored the impact of progressive taper enlargement on buccal root canals of three‐rooted maxillary molars using micro‐CT and found that an increased taper significantly enhances dentin removal. This finding agrees with Al‐Dhbaan et al. [19] who compared the shaping ability of PTG and WaveOne Gold NiTi rotary files in various canal configurations and demonstrated that both systems effectively shaped canals, albeit with differences influenced by canal anatomy. Although these studies [19, 32] did not use mandibular incisors, they collectively underline the importance of selecting rotary files that strike a balance between efficient shaping and optimal dentin preservation. This fact reinforces the relevance of the VDWR design in minimizing tissue loss while achieving effective canal preparation.

Furthermore, Dablanca‐Blanco et al. [33] underscored the necessity for endodontists to be well versed in the characteristics of different rotary files since this knowledge is essential for achieving optimal treatment outcomes. Taken together, these studies demonstrate that understanding rotary file design and taper geometry is crucial for enhancing canal shaping efficacy while preserving dentin integrity, ultimately contributing to the success of endodontic therapy. Adapting endodontic strategies to individual canal morphologies will not only improve shaping results but also help prevent complications related to excessive dentin removal.

Although micro‐CT provides a highly accurate assessment of dentin removal, this in vitro design does not account for functional loading, restorative procedures, or stresses applied to the pericervical region during mastication. Therefore, the volumetric differences observed between PTG and VDWR should not be interpreted as evidence of potential differences in clinical fracture resistance or long‐term prognosis. These findings reflect the mechanical behavior of the instruments under controlled laboratory conditions and do not necessarily translate to clinical performance.

In clinical terms, mandibular incisors rarely fail due to loss of pericervical dentin. Instead, other factors such as missed anatomy, inadequate disinfection, or coronal leakage are more commonly associated with treatment failure. The present findings agree with previous studies such as that of Silva et al. [14] that emphasized the importance of dentin preservation. Although our findings characterize the shaping behavior of PTG and VDWR, their relevance to long‐term prognosis in anterior teeth should be viewed with caution.

## 5. Conclusion

Within the limitations of this study, VDWR removed less dentin than PTG in oval canals. Despite the taper disparities between systems that may confound direct comparison, both systems maintained canal geometry effectively, and neither was able to fully prepare the oval mandibular incisors.

## Disclosure

We declare that our roles as researchers are independent and committed to the primary interest of defending rights and ensuring the safety of the research participant(s) in accordance with Resolution 466/12 and other ethical guidelines for research involving human beings.

## Conflicts of Interest

The authors declare no conflicts of interest.

## Funding

This work was supported by Coordenação de Aperfeiçoamento de Pessoal de Nível Superior, (10.13039/501100002322) 001.

## Data Availability

Data sharing not applicable to this article as no datasets were generated or analyzed during the current study.
